# Cleavage and polyadenylation factors are potential regulators of adipogenesis

**DOI:** 10.1186/s13104-024-06908-3

**Published:** 2024-09-02

**Authors:** Salwa Mohd Mostafa, Claire Moore

**Affiliations:** Graduate School of Biomedical Sciences, Department of Developmental, Molecular, and Chemical Biology, Tufts University School of Medicine, Boston, MA 02111 USA

**Keywords:** Cleavage and polyadenylation, Adipogenesis, 3T3-L1

## Abstract

**Objective:**

Alternative polyadenylation (APA) is a co-transcriptional process that leads to isoform diversity in the 3’ ends of mRNAs. APA is known to occur during differentiation, and its dysregulation is observed in diseases like cancer and autoimmune disorders. It has been previously reported that differentiation of 3T3-L1 cells to adipocytes leads to an overall lengthening of mRNAs, but the proteins involved in this regulation have not been identified. The expression levels of subunits of the cleavage and polyadenylation (C/P) complex can regulate the choice of poly(A) site, which in turn can affect different cellular activities. In this paper, we studied the change in levels of C/P proteins during 3T3-L1 differentiation.

**Results:**

We observed that while the RNA expression of these proteins is unchanged during differentiation, the protein levels of some subunits do change, including a decrease in levels of CPSF73, the nuclease that cuts at the poly(A) site. However, overexpression of CPSF73 alone does not affect the efficiency and rate of differentiation.

**Supplementary Information:**

The online version contains supplementary material available at 10.1186/s13104-024-06908-3.

## Introduction

Adipogenesis is a finely regulated process for differentiation of preadipocytes to adipocytes. Dysregulation of this process can lead to health complications, such as the development of obesity and obesity-related disorders [1]. Therefore, understanding the regulation of adipogenesis at the molecular level would contribute to designing therapeutics. Cleavage and polyadenylation (C/P) is an essential mRNA processing step in which transcripts are cleaved at a specific site, followed by addition of a poly(A) tail that is important for mRNA stability, export, and translation. C/P is brought about by four major complexes – CPSF (cleavage and polyadenylation specificity factor), CSTF (cleavage stimulation factor), CFIm (mammalian cleavage factor I) and CFIIm (mammalian cleavage factor II), and additional core factors including symplekin, polyA polymerase (PAP), nuclear poly(A) binding protein (PABPN1) and the C-terminal domain (CTD) of the largest subunit of RNA polymerase II [2]. These proteins are imperative to the transcription termination step and play important roles in alternative polyadenylation (APA) – a regulatory step that leads to isoform diversity due to differential usage of polyadenylation (pA) sites [2]. APA contributes to changes in cell state during disease or normal differentiation and development. For example, our group has shown that changes in protein levels of C/P factors during macrophage differentiation are associated with significant shifts in pA site usage and that CSTF64, a protein in the CSTF complex with known roles in APA, directly regulates the process [3]. Previously, Hoque et al. found that there is an overall lengthening of 3’-UTR due to APA during differentiation of 3T3-L1 preadipocytes [4]. However, changes in the expression of C/P factors during adipogenesis are still unknown. Therefore, we aimed to study the changes of C/P factors during adipogenesis using 3T3-L1 as the model system.

## Methods

### Cell culture and Oil Red O staining

3T3-L1 cells were cultured in maintenance media (Dulbecco’s Modified Eagle Medium (DMEM - high glucose) containing 10% fetal bovine serum (FBS) and antibiotics (100 U/ml penicillin, 100 ug/ml streptomycin)). Cells were grown at 37 °C with 5% CO_2_. Cells were passaged every 3 days to prevent cell confluency over 80%. For differentiation, cells were plated at a density of 1.0 × 10^4^ cells/cm^2^ and grown till they reached 100% confluency. Cells were then maintained for two additional days to allow for contact inhibition. On day 0, the cells were induced to differentiate using differentiation media (maintenance media supplemented with 0.5 mM 3-isobutyl-1-methylxanthine, 1 μm dexamethasone, 5 ug/mL insulin, and 1 μm rosiglitazone). On day 3, the differentiation media was replaced with insulin media (maintenance media containing 5 ug/mL insulin). On day 5, insulin media was replaced with maintenance media and cultured for 2 additional days before being harvested for protein or RNA.

3T3-L1 cells containing control (3T3-L1-C) and CPSF73-overexpressing (3T3-L1-OE) vectors were generated previously in the lab [5]. Cells were grown and differentiated in maintenance media, differentiation media and insulin media as described above, except with the addition of 1.5 ug/ml of doxycycline (Dox) when the cells were plated for the differentiation experiment. The Dox-containing media were replenished every other day. Cells on Day 0 were subjected to differentiation media for 2 days, insulin media for 2 days, and maintenance media for 4 days before being harvested for protein and RNA and stained with Oil Red O staining.

For Oil Red O staining, cells at Day 0 and Day 8 were washed twice with phosphate-buffered saline (PBS) and then fixed with 4% formaldehyde for one hour at room temperature. Then, the cells were washed once with PBS, once with 60% isopropanol, and dried. A 0.5% stock solution of Oil Red O (Sigma–Aldrich, Sigma O0625) in isopropanol was prepared and filtered through a 0.2-um filter. A fresh working solution was prepared by mixing the stock solution with distilled water in a 6:4 ratio, incubating it for 20 min at room temperature, and re-filtering through a 0.2-um filter. The working solution was then added to the fixed cells and incubated at room temperature for 1 h. The cells were then washed extensively with distilled water, dried and photographed.

### RNA extraction and RT-qPCR

Cells were lysed with Trizol (Thermo Fisher Scientific, 15-596-018) and RNA was extracted using the Zymo Direct-Zol RNA Miniprep kit (Zymo Research, R2050). One ug of RNA was then reverse transcribed to cDNA using NEB LunaScript^®^ RT SuperMix kit (New England Biolabs, M3010L), and qPCR was carried out using NEB Luna^®^ Universal qPCR Master Mix (New England Biolabs, M3003L) and primers listed in Supplementary Table 1. Expression of adipogenesis markers were normalized to that of mouse *Rpl13a* and the qPCR results were quantified using the ddCt method.

### Western blotting

Cells were lysed with RIPA buffer (150 mM sodium chloride, 50 mM Tris-HCl (pH 7.4), 1% NP-40, 0.1% SDS, 5 mM EDTA, 0.1% sodium deoxycholate, 1 mM Dithiothreitol, 10 mM sodium fluoride, 200 μm sodium orthovanadate, 1 mM sodium pyrophosphate, 10 mM B-glycerophosphate) with protease inhibitor cocktail (Bimake, B14001; Thermo Fisher Scientific, PI78425). Cells in RIPA buffer were incubated on ice for 15 min and then homogenized by vortexing. The lysates were cleared by centrifugation at 12,000 x g for 15 min at 4 °C. Protein quantification was done by the BCA assay (Thermo Fisher Scientific, A53226).

The lysates were prepared for western blotting with the addition of 4X SDS loading buffer with 355 mM β-mercaptoethanol and heated to 95 °C for 5 min. Then, 25–50 mg of lysate was resolved on a 10% Bis-Tris gel and transferred to PVDF membranes. Total protein staining was done using Revert Total Protein Stain (LI-COR, 926-11021). Using molecular weight markers as guidelines, blots were cut into strips for probing with different antibodies. Membranes were then blocked for 5 min using EveryBlot Blocking Buffer (Bio-Rad, 12,010,020) or for 30 min using 5% milk in 1x TBST (Tris Buffered Saline Buffer with Tween 20). Primary antibodies (Supplementary Table 2) were diluted in 1% milk in 1x TBST, EveryBlot or only 1x TBST and incubated with membrane overnight at 4 °C. Membranes were washed three times in 1x TBST and incubated with HRP (HorseRadish Peroxidase)-labeled secondary antibodies for 1 h at room temperature. A Syngene imager was used to capture the chemiluminescence signal of proteins and ImageJ was used to quantify the band intensities. A LI-COR Odyssey CLx was used to capture the total protein stain which was then quantified using Image Studio (version 5.5). Images of the protein-stained gels and uncropped blots are presented in Supplemental Figs. 1 and 2.

### Differential gene expression analysis

For analysis of differential gene expression during 3T3-L1 differentiation, we used the undifferentiated Day 0 (GSM3728574, GSM3728575, GSM3728576) and differentiated Day 8 (GSM3728580, GSM3728581, GSM3728582) samples from the publicly available GEO dataset GSE129957 [6]. The raw sequence reads of the samples were processed to remove adapters using Cutadapt [7] (v2.8) and the quality of these trimmed reads was checked using FastQC [8] (v0.11.8) and MultiQC [9] (v1.7.0). The reads were then aligned using STAR [10] (2.6.1d) to mouse mm10 genome. Aligned reads were then quantified using featurecounts [11] (v1.6.3) and log2-fold changes were calculated with DESeq2 [12](1.40.2) using default parameters. A fold change of 1 and an adjusted p-value of less than 0.05 were applied to select differentially expressed genes.

## Results

### Levels of some cleavage and polyadenylation factors change during 3T3-L1 differentiation

**Fig. 1:**
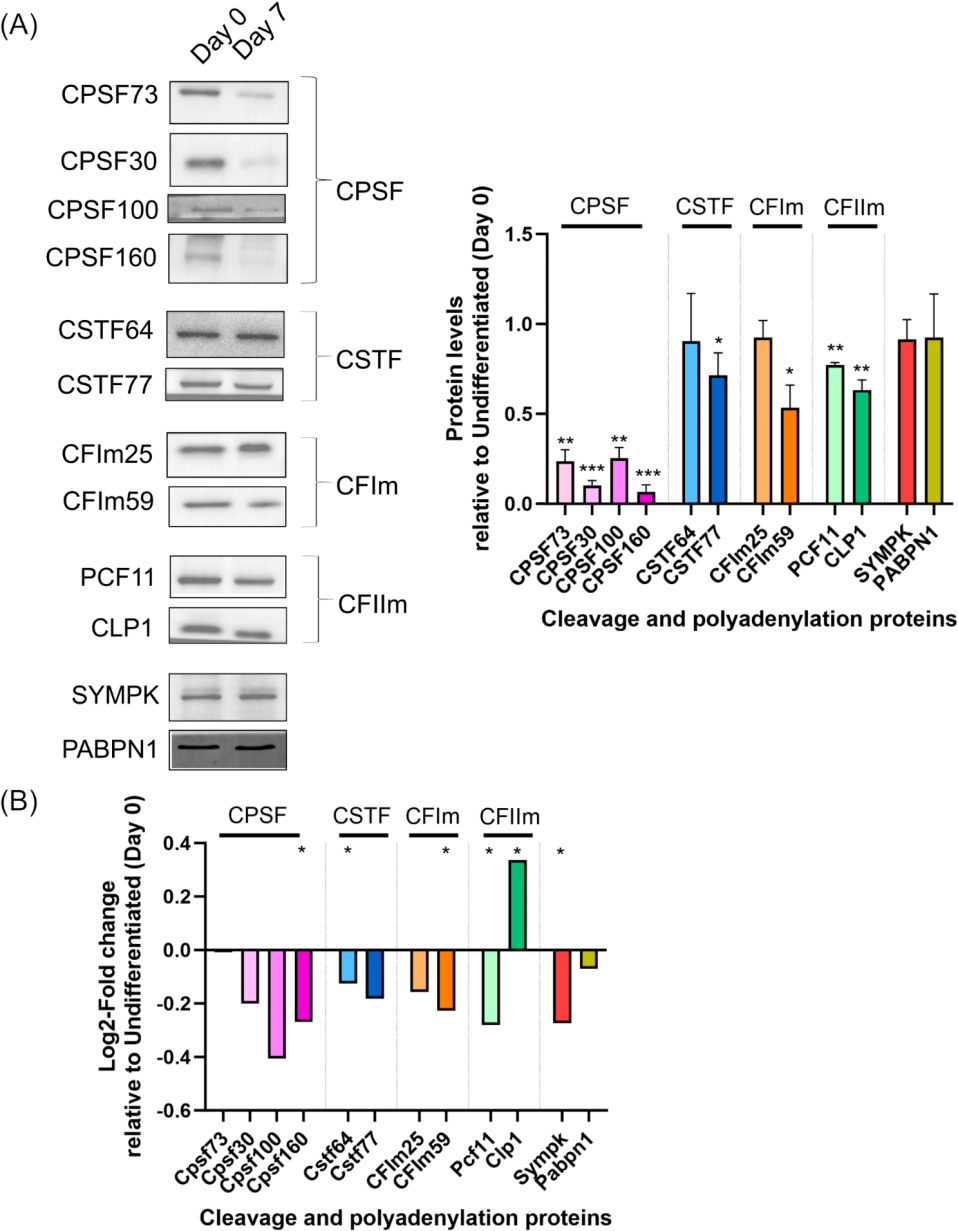
Protein levels of cleavage and polyadenylation factors change during 3T3-L1 differentiation. (**A**) Representative cropped blots of protein levels of C/P factors from each complex are shown, along with quantifications (*n* = 3, mean ± SD) of protein levels at Day 7 relative to Day 0, where values above 1 indicate increased levels and values below 1 indicate decreased levels during differentiation. The protein levels were normalized to total protein levels. Significance testing was done using student’s unpaired two-tailed t-test with Welch’s correction, with p-values less than 0.05 considered significant and denoted with an asterisk. Full-length total-protein stained gels and uncropped blots are presented in Supplementary Figure S1. (**B**) Determination of change in mRNA expression of C/P factors during differentiation with threshold of log2-fold change of 1 and p-adjusted value < 0.05. P-adjusted values less than 0.05 are labelled with an asterisk.

We analyzed the protein levels of representative subunits of the C/P complex between undifferentiated and differentiated 3T3-L1 cells and found changes in some of the tested C/P subunits (Fig. 1A). Multiple proteins decrease during differentiation, especially all of the tested CPSF proteins, which show the strongest decrease. CTSF77, CFIm59 and CFIIm (PCF11 and CLP1) also decrease in protein levels during 3T3-L1 differentiation. CSTF64, CFIm25, SYMPK and PABPN1 do not have remarkable differences in protein levels between undifferentiated and differentiated cells. Interestingly, the expression of mRNAs encoding the affected proteins did not change to an extent that would account for the observed decrease in protein levels of tested C/P factors (Fig. 1B).

### Overexpression of CPSF73 alone does not the change efficiency or rate of 3T3-L1 differentiation

**Fig. 2:**
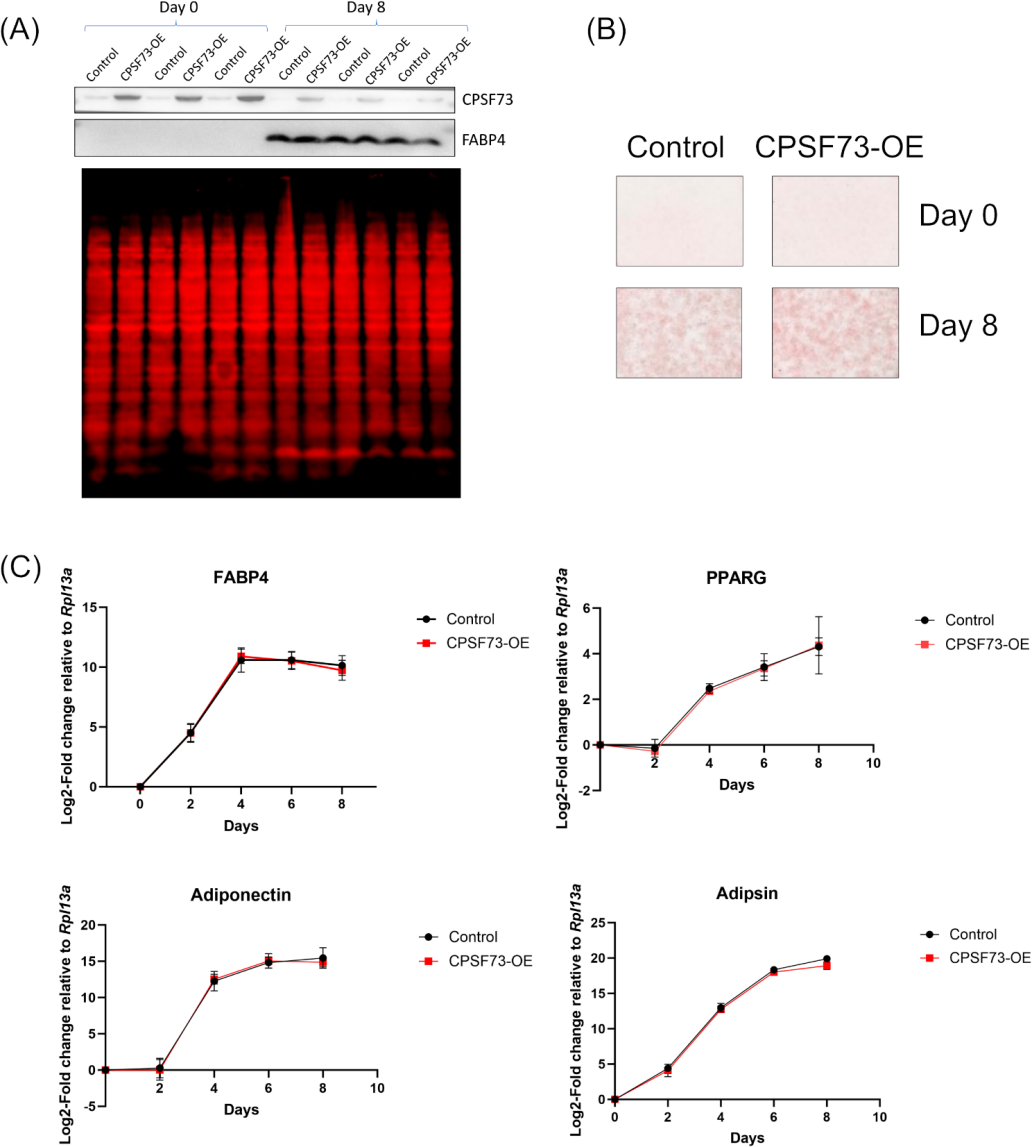
Overexpression of CPSF73 alone is not sufficient to affect differentiation efficiency. (**A**) CPSF73 is overexpressed in both undifferentiated and differentiated adipocytes compared to its control. Three replicates from Day 0 and from Day 8 of differentiation are shown. The FABP4 marker indicates successful differentiation, and the total protein stain (bottom panel) serves as loading control. Full-length total-protein stained gels and uncropped blots are presented in Supplementary Figure S2. (**B**) Oil Red O staining of Dox-induced control and CPSF73-ovexpressing 3T3-L1 cells at Day 0 and Day 8 of differentiation does not show a change in differentiation efficiency due to overexpression of CPSF73. (**C**) Overexpression of CPSF73 does not change the rate of 3T3-L1 differentiation. Expression of mRNAs of adipogenesis markers normalized to *Rpl13A* are shown relative to Day 0 of differentiation.

Considering the observed large, significant decrease in protein levels of CPSF73 during adipogenesis, its central function as the nuclease of the processing complex, and our previous work showing that it was needed to maintain the committed state of preadipocytes [5], we investigated whether the differentiation of 3T3-L1 cells is affected when CPSF73 is overexpressed. We used Control- and CPSF73-overexpressing 3T3-L1 cells previously generated in the lab [5] and induced CPSF73 overexpression when cells were plated for differentiation, so that CPSF73 was overexpressed in both undifferentiated (Day 0) and differentiated (Day 8) cells (Fig. 2A). Even though CPSF73 levels in the CPSF73-overexpressing cells decreased at Day 8, the levels were still higher than that in the control cells. Using Oil Red O staining of fixed undifferentiated and differentiated cells, we did not see a remarkable change in differentiation efficiency upon overexpression of CPSF73 (Fig. 2B). Since the overexpression of CPSF73 could be affecting the rate at which cells achieve the differentiated state without necessarily affecting the overall differentiation efficiency, we tested the mRNA levels of adipogenesis markers *Fabp4*, *Ppparg*, Adipsin (*Cfd*) and Adiponectin (*Adipoq*) [5] across five timepoints during differentiation (Days 0, 2, 4, 6 and 8). However, the expression of markers during the process was not affected by CPSF73 overexpression (Fig. 2C).

## Discussion

C/P factors play important roles in the 3’-end processing of pre-mRNAs to mRNAs and are implicated in diseases like cancer [13]. Changes in their protein levels can directly regulate APA [2] and therefore, studying their changes during 3T3-L1 differentiation identifies potential mechanisms to regulate gene expression during adipogenesis, for example, in the lengthening of genes due to APA during 3T3-L1 differentiation [4]. We have shown that protein levels of multiple C/P factors change during 3T3-L1 differentiation without changes in RNA expression. This may be due to changes in translational efficiency or protein stability [14–16]. For example, miRNAs could block protein translation without mRNA degradation and thus affect protein levels without affecting RNA levels.

Of special note is the decrease in protein levels of the tested CPSF subunits. These proteins are required for cleavage of the pre-mRNA and to direct AAUAAA-dependent poly(A) tail addition [2]. Therefore, decrease in the levels of CPSF factors could lead to changes in cleavage efficiency of pre-mRNAs or contribute to APA changes during differentiation, and therefore regulate adipogenesis. However, even though the levels of CPSF73 are substantially decreased in differentiated cells compared to undifferentiated cells, we found no effects of overexpressing CPSF73 on adipogenesis.

### Limitation

One limitation of this study is that we may not have been able to overexpress CPSF73 at a level high enough to impair differentiation. In addition, we have only tested the effect of CPSF73 overexpression on overall efficiency of differentiation and the rate at which cells reach the differentiated state without testing other potential effects on (a) the release of secretory factors like extracellular matrix proteins during adipogenesis [17], (b) lipid droplet dynamics [18] or fatty acid [19] and protein composition [20] of lipid droplets, which could have implications in browning of white adipose tissue [21] and the development of lipid disorders [22], and (c) metabolite concentrations at different timepoints during differentiation which are relevant in understanding obesity and related disorders like Type 2 diabetes mellitus [23].

Moreover, we have not tested the effects of overexpressing other components of the C/P complex individually or in combination, which could have a more substantial effect on 3T3-L1 differentiation. For example, CSTF77 is known to interact with CPSF160 and potentially enable CPSF-CstF cooperative RNA binding during the assembly of the C/P machinery [2]. CFIm59 also decreases during 3T3-L1 differentiation and may therefore be a potential regulator. The CFIm complex consists of a CFIm25 dimer and a dimer of CFIm59 or CFIm68. While CFIm59 was thought to primarily have a redundant function with CFIm68, it was shown to have a distinct effect on APA of *PTEN* gene in the mouse fibroblast cell line NIH3T3 [24]. Although proteins of the CFIIm complex (PCF11 and CLP1) do not undergo as large a decrease in levels as CPSF factors, their decrease during adipogenesis might affect pre-mRNA cleavage efficiency or the assembly of C/P machinery [2, 25–27]. Interestingly, CSTF64 and PABN1, two well-known APA regulators [2], are not altered in levels.

In summary, this paper reveals important changes in levels of C/P factors during adipogenesis and provides the basis for further experiments to characterize the roles of C/P factors in the process.

### Electronic supplementary material

Below is the link to the electronic supplementary material.

Supplementary Material 1

## Data Availability

Differential gene expression analysis was done on samples GSM3728574, GSM3728575, GSM3728576, GSM3728580, GSM3728581, and GSM3728582 from the publicly available GEO dataset GSE129957.

## Abbreviations

APA: Alternative polyadenylation
C/P: Cleavage and polyadenylation
pA: poly(A)
CPSF: Cleavage and Polyadenylation Specificity Factor
CSTF: Cleavage Stimulation Factor
CFIm: Mammalian Cleavage Factor I
CFIIm: Mammalian Cleavage Factor II
CTD: C-terminal Domain
