# Supplementary material for: Cleavage and polyadenylation factors are potential regulators of adipogenesis
Source: BMC Res Notes. 2024 Sep 2;17:242. doi: 10.1186/s13104-024-06908-3 (PMC11370009; doi:10.1186/s13104-024-06908-3)
Supplement: Supplementary file 1 — Supplementary Material 1 [file 13104_2024_6908_MOESM1_ESM.pdf]

Supplementary materials

Supplementary Figure S1: Uncropped blots of proteins shown in Figure 1. As described in the Methods, cell extract was resolved on a 10% Bis-Tris gel and transferred to PVDF membranes. Before blocking, the blot was stained for total protein (top image), with marker lanes labeled as “M”. After blocking, but before incubation with primary antibody, the full blot was cut into sections using protein markers as guidelines to allow detection of multiple proteins from the same sample. The uncropped raw images for each protein blot are shown in the bottom images and the blots are aligned to the lanes used. The molecular weights of standard protein size markers are indicated on the left side of each blot and the names of proteins are included below or to the side of the blot. The brightness and contrast were not modified from the raw images. For blots that contain more than one pair of lanes of Day 0 (undifferentiated) and Day 7 (differentiated) samples, the pair of lanes shown in Figure 1A is indicated by a red line above the blot.

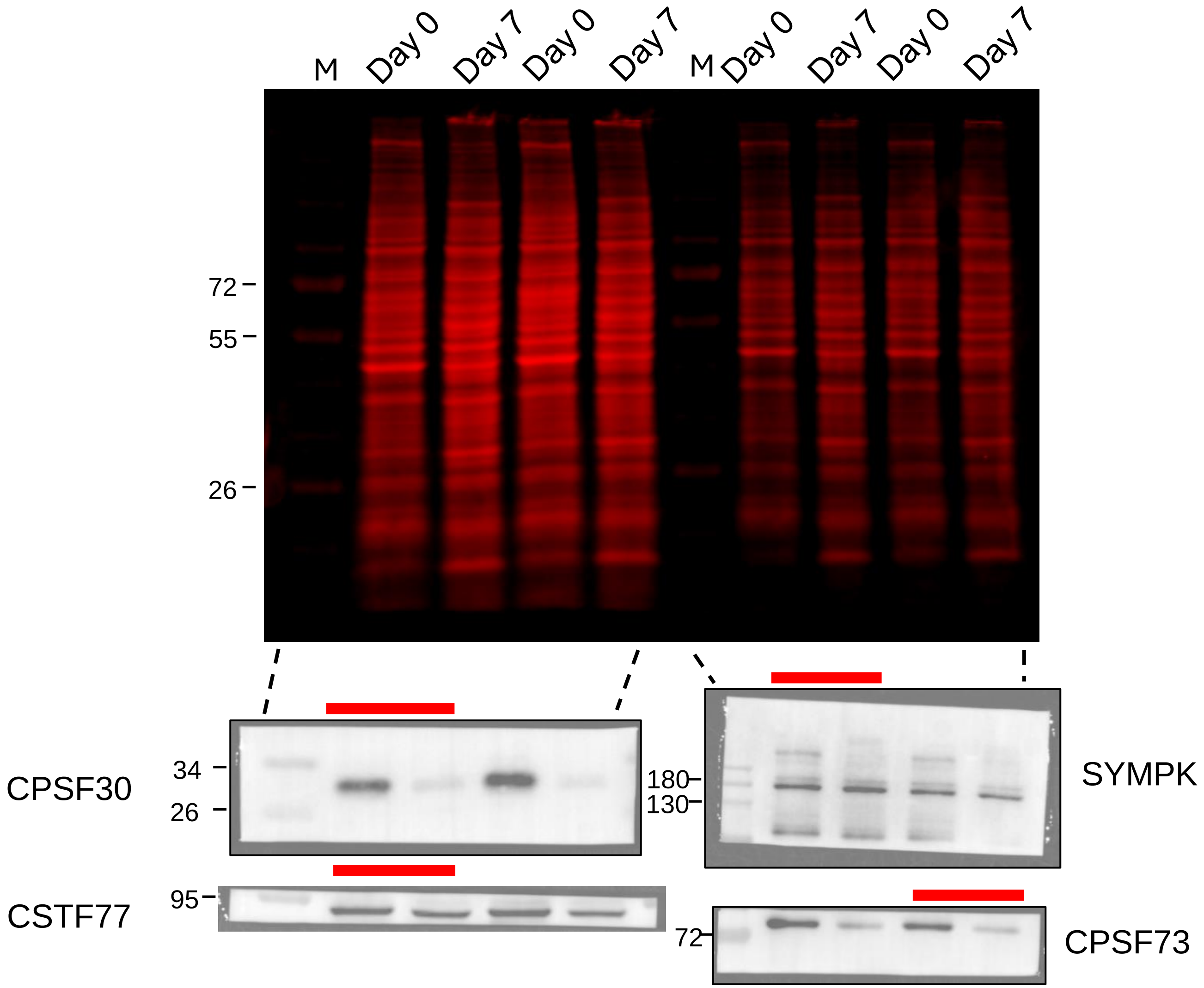

M
Day 0
Day 7
Day 0
Day 7
M
Day 0
Day 7
Day 0
Day 7
72
55
26
CPSF30
34
26
180
130
SYMPK
CSTF77
95
72
CPSF73

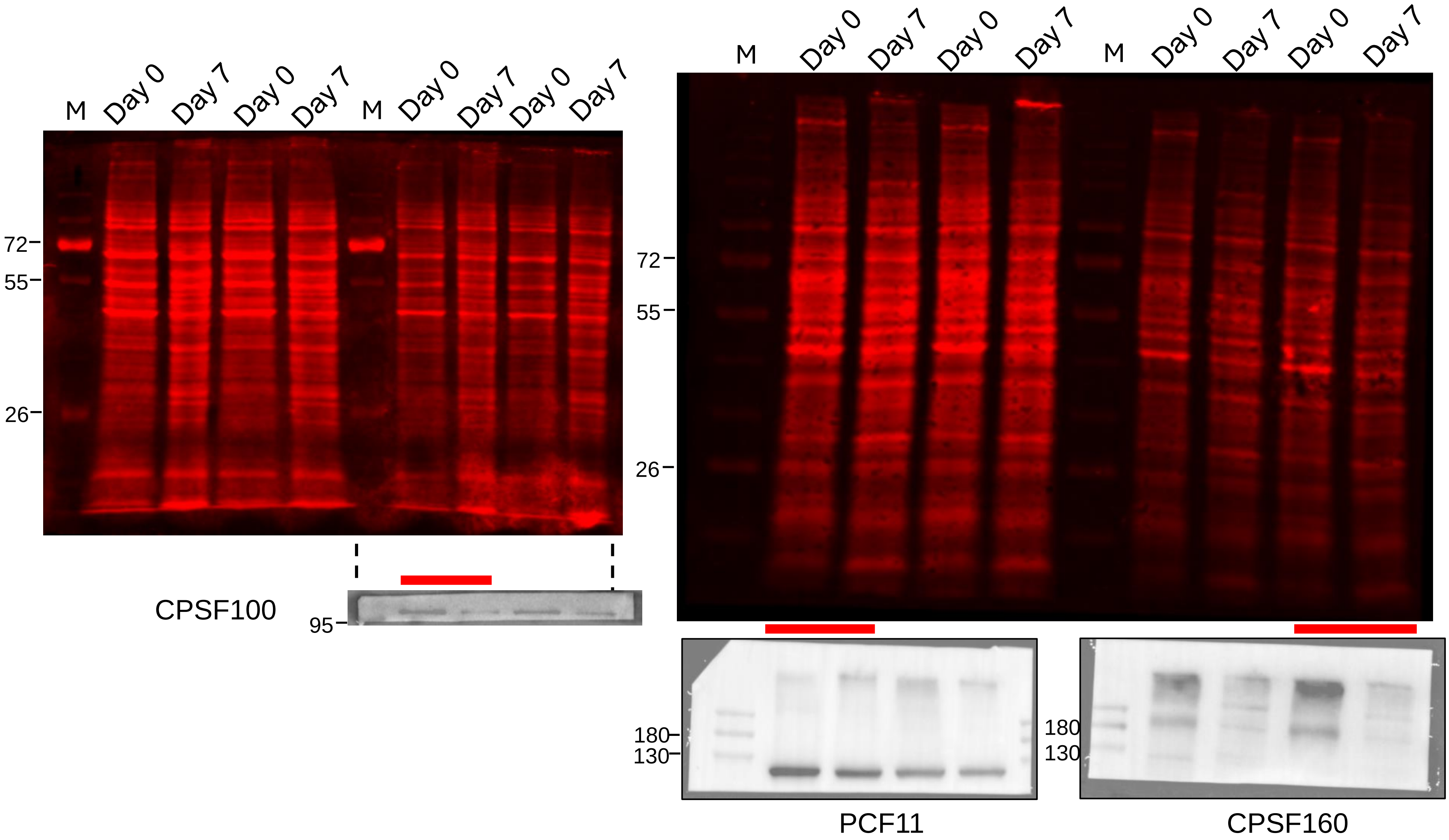

M
Day 0
Day 7
Day 0
Day 7
M
Day 0
Day 7
Day 0
Day 7
72
55
26
CPSF100
95
M
Day 0
Day 7
Day 0
Day 7
M
Day 0
Day 7
Day 0
Day 7
72
55
26
180
130
PCF11
180
130
CPSF160

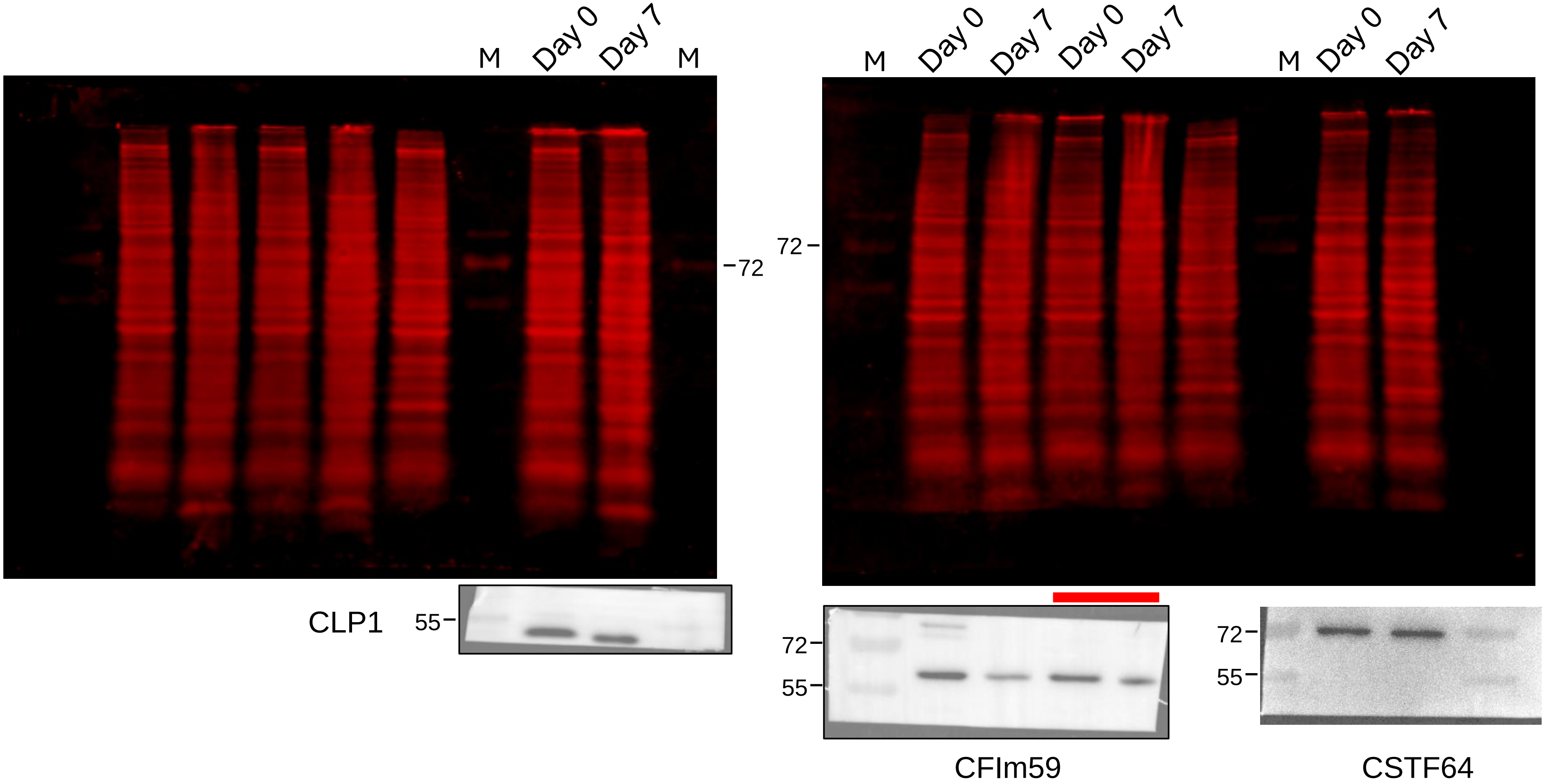

M
Day 0
Day 7
M
72
CLP1
55
M
Day 0
Day 7
Day 0
Day 7
M
Day 0
Day 7
72
55
CFIm59
CSTF64

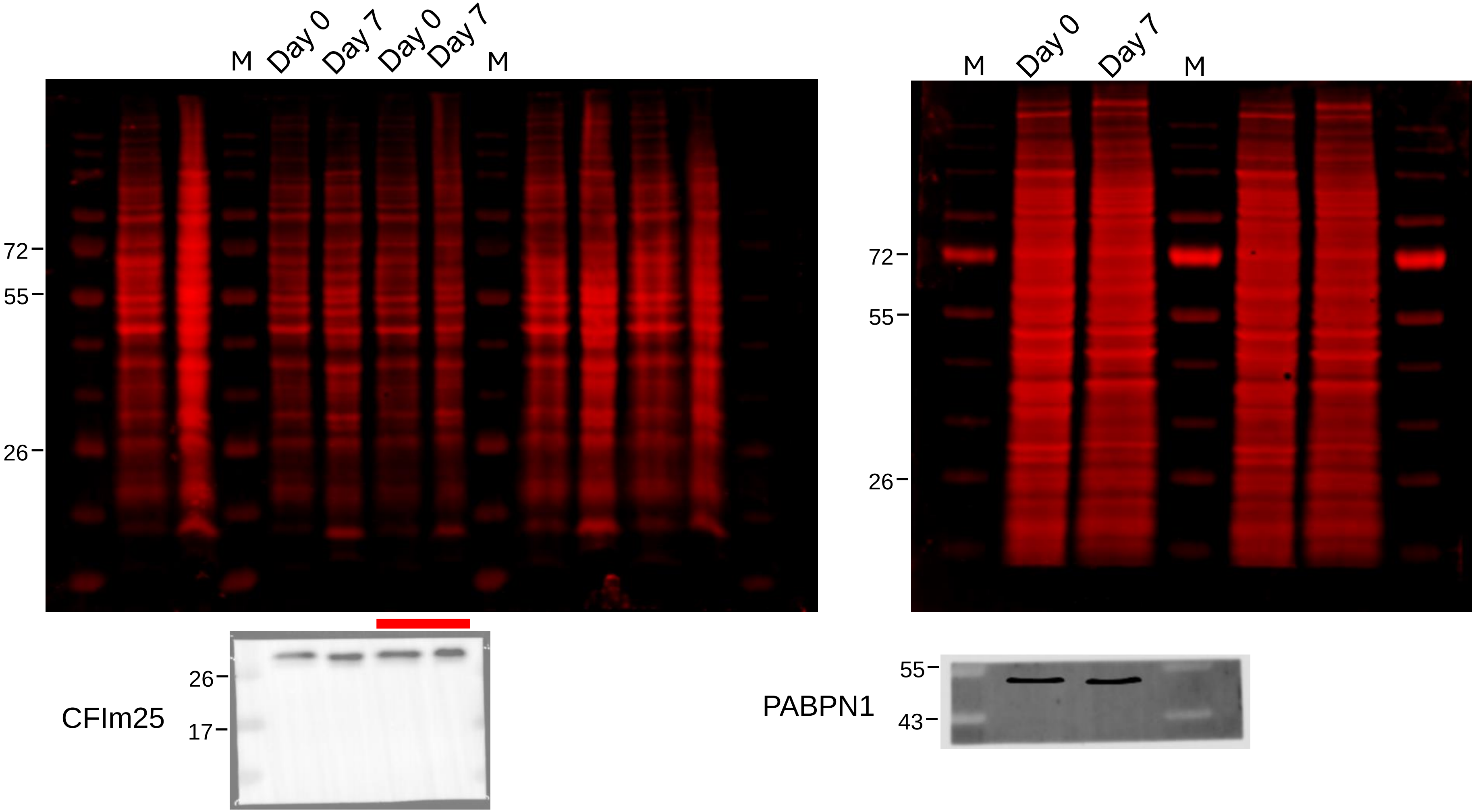

M
Day 0
Day 7
Day 0
Day 7
M
72
55
26
CFIm25
26
17
M
Day 0
Day 7
M
72
55
26
PABPN1
55
43

Supplementary Figure S2: Uncropped blots of proteins shown in Figure 2. As described in the Methods, cell extract was resolved on a 10% Bis-Tris gel and transferred to PVDF membranes. Before blocking, the blot was stained for total protein (top image), with marker lanes labeled as “M”. After blocking, but before incubation with primary antibody, the full blot was cut into sections using protein markers as guidelines to allow detection of multiple proteins from the same sample. The uncropped raw images for each protein blot are shown in the bottom images and the blots are aligned to the lanes used. The molecular weight of standard protein size markers is indicated on the left side of each blot and the names of proteins are included on the right side of the blot. The brightness and contrast were not modified from the raw images.

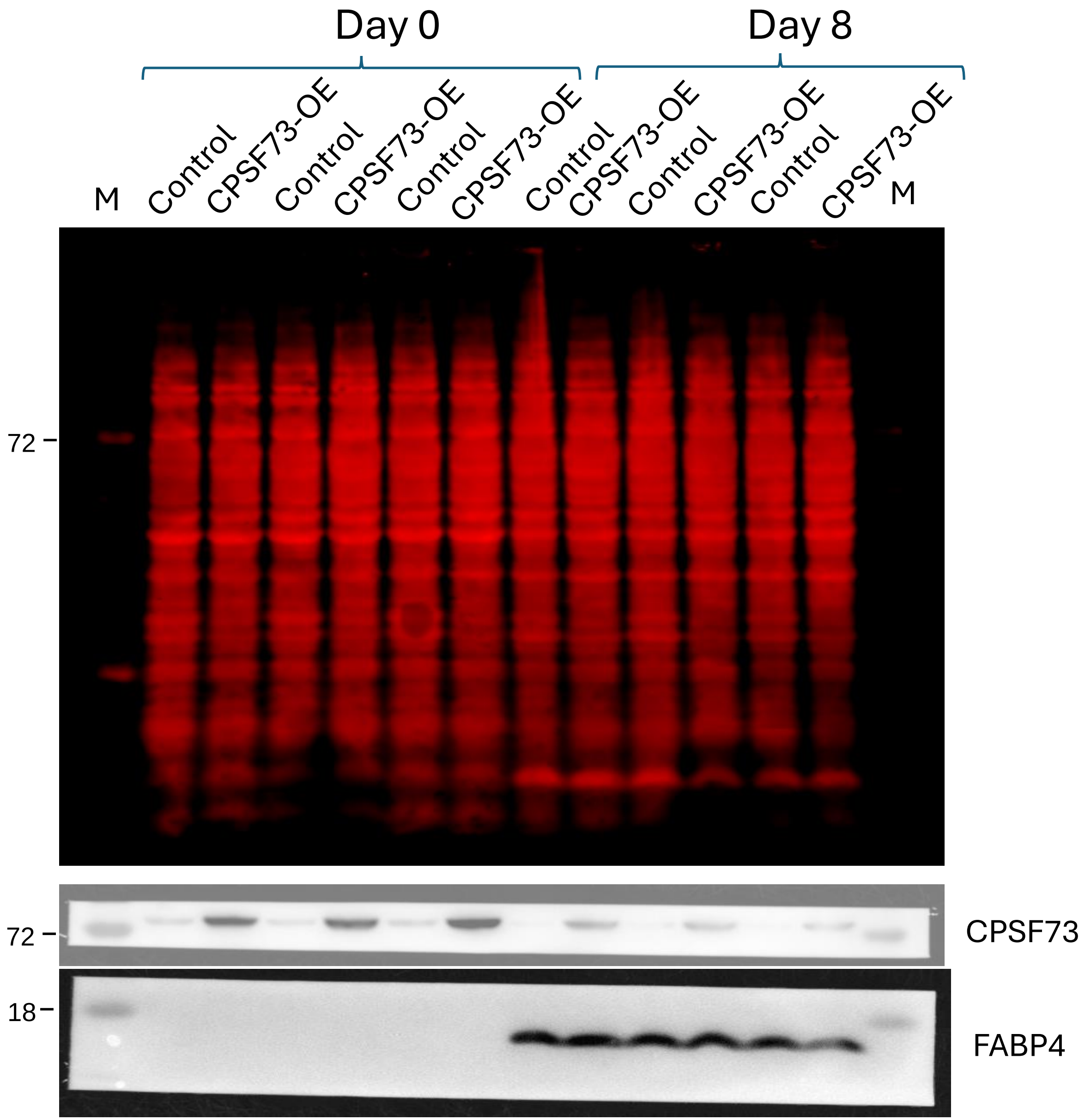

Day 0
Day 8
M
Control
CPSF73-OE
Control
CPSF73-OE
Control
CPSF73-OE
Control
CPSF73-OE
Control
CPSF73-OE
Control
CPSF73-OE
M
72
72
18
CPSF73
FABP4

Supplementary tables

Primers	Sequence
Fabp4 Forward	AAGGTGAAGAGCATCATAACCCT
Fabp4 Reverse	TCACGCCTTTCATAACACATTCC
Adipoq Forward	TGTTCCTCTTAATCCTGCCCA
Adipoq Reverse	CCAACCTGCACAAGTTCCCTT
Cfd Forward	CATGCTCGGCCCTACATGG
Cfd Reverse	CACAGAGTCGTCATCCGTCAC
Pparg Forward	CTCCAAGAATACCAAAGTGCGA
Pparg Reverse	GCCTGATGCTTTATCCCCACA
Rpl13a Forward	CTGCTCTCAAGGTTGTTCGGCT
Rpl13a Reverse	CCTTCCGTTTCTCCTCCAGAGT

Supplementary table 1: List of primers

Protein	Antibody source	Blocking buffer
CPSF73	Santa Cruz Biotechnology (SCBT), sc-393001	5% milk in 1x TBST
CPSF30	Novus Biologicals, NB100-79827	EveryBlot Blocking Buffer
CPSF100	SCBT, sc-165983	5% milk in 1x TBST
CPSF160	SCBT, sc-166281	5% milk in 1x TBST
CSTF64	Bethyl Laboratories, A301-092A	5% milk in 1x TBST
CSTF77	SCBT, sc-376575	5% milk in 1x TBST
CFIm25	Bethyl Laboratories	5% milk in 1x TBST
CFIm59	SCBT, sc-393880	5% milk in 1x TBST
PCF11	SCBT, sc-514158	5% milk in 1x TBST
CLP1	Proteintech, 14746-1-AP	5% milk in 1x TBST
SYMPK	SCBT, sc-398897	5% milk in 1x TBST
PABPN1	Abclonal, A6041	5% milk in 1x TBST
FABP4	SCBT, sc-271529	5% milk in 1x TBST

Supplementary table 2: List of antibodies
